# Clinical significance of treatment delay in status epilepticus

**DOI:** 10.1186/1865-1380-6-6

**Published:** 2013-02-27

**Authors:** Jonas Hillman, Kai Lehtimäki, Jukka Peltola, Suvi Liimatainen

**Affiliations:** 1 Medical School, University of Tampere, Tampere, Finland; 2 Department of Neurosurgery, Tampere University Hospital, Tampere, Finland; 3 Department of Neurology and Rehabilitation, Tampere University Hospital, P.O. Box 2000, 33521, Tampere, Finland; 4 Department of Emergency Medicine Acuta and Department of Neurology and Rehabilitation, Tampere University Hospital, Tampere, Finland

**Keywords:** Status epilepticus, Prolonged seizure, Emergency, Treatment delay, Antiepileptic drug

## Abstract

**Background:**

Status epilepticus (SE) is a medical emergency that requires immediate action. The clinical and demographic features of SE are known to be highly variable. The objective of this study was to analyze the effect of treatment delays on patient recovery and different clinical factors that are important in the determination of the acute prognosis in SE.

**Methods:**

This population-based study included 109 consecutive visits of patients with the diagnosis of SE in the emergency department (ED) of Tampere University Hospital. The clinical features of SE were compared with the discharge condition.

**Results:**

The treatment delays were long; in half of the patients, the delay for paramedic arrival was over 30 min, and in one-third of the cases, the delay was over 24 h. ED patients who had less than 1 h of delay before the administration of an antiepileptic drug (AED) had better outcomes compared to patients with a greater than 1 h delay (*p* < 0.05). The two major etiologies for the SE were cerebrovascular disease and alcohol misuse. A good immediate outcome was found in 46% of the patients. Epileptiform activity on the EEG, a history of epilepsy or SE, presence of cardiovascular disease, and alcohol misuse were associated with a poor outcome.

**Conclusions:**

The results of this study emphasize the importance of an urgent response by emergency services and proper recognition of atypical phenotypes of SE.

## Background

Status epilepticus (SE) is a medical emergency that requires rapid diagnosis and treatment. Both clinical studies and empirical experience indicate a variation in treatment practices and treatment delays in prolonged seizures. A Finnish study of 157 children strengthened the previously known association between treatment delays and outcomes [1]. Earlier studies have shown the impact of age on the outcome of SE, children having better chances of recovery [2]. The etiology of SE is also associated with prognosis [3]. Acute symptomatic causes (= acute cerebral insults with seizures) and cerebrovascular disease as the etiology worsen the prognosis [4].

In this study, we evaluated treatment delays, different clinical features of the SE, and the use of antiepileptic drugs (AED) for prolonged seizures and their association with patient recovery. All adult patients with prolonged seizures in the Pirkanmaa Hospital District (population of 440,000), except those patients already hospitalized in our hospital, are treated in our emergency department (ED). For this reason, the present study represents population-based treatment practices of EDs in Finland.

## Methods

### Patients

This study was a retrospective, population-based study of 109 consecutive visits (100 patients) of adult patients with a diagnosis of status epilepticus (SE) (ICD10 code G41.0–41.9) who were treated in the ED of Tampere University Hospital between 1 May 2007 and 23 November 2009. We aimed to obtain a maximal number of patients to explore the clinical treatment practices. The data were collected from medical records. Children were excluded from the study because there is a separate pediatric ED in our hospital. The medical records of all the patients that met the diagnosis of SE were reviewed to identify the clinical features that affected the current episode. We used an established definition of SE: a seizure lasting ≥ 30 min or recurring seizures without full recovery between seizures [5,6]. These previous studies suggest SE lasting over 30 min increases the mortality rate [5,6]. The study protocol followed the guideline of Declaration of Helsinki and STROBE statement. The study was approved by the Ethics Committee of Tampere University Hospital.

### Treatment delay

The treatment delay was calculated based on the time of the beginning of the seizure (if known), the arrival of paramedics, the initiation of AED treatment out of hospital and in the ED, and the arrival at the ED. In patients for whom the beginning of the seizure was unknown, we estimated the duration based on available data from eyewitnesses, relatives, and nursing personnel.

### Co-morbidity

Previous disorders, such as previous brain disease [e.g., epilepsy, dementia, stroke, tumor, and central nervous system (CNS) infection], psychiatric disease, cardiovascular disease, diabetes, traumatic episodes, misuse of alcohol or drugs, and other diseases, as well as the history of SE, were recorded.

### Assessment of neurological condition

#### Neurological parameters

*The following parameters were recorded:* convulsions in any part of the body; psychomotor slowness; disorientation; confusion; aphasia; pupillary reflex; unilateral sensorimotor deficit in the face, arm or leg; deviation of the head and eyes; neglect; nystagmus; and other findings. The Glasgow Coma Scale (GCS) score was recorded.

### Seizure classification

The seizure type was categorized as unclassified generalized, secondary generalized, complex partial, and sim ple partial according to the International League Against Epilepsy (ILAE) classification [7]. The seizures were classified as secondary generalized (SGSE) based on the epilepsy history, seizure semiology, and neurological condition when there were signs of focal onset of the seizure or a focal lesion in the neuroimaging that correlated with the seizure type. In the rest of the patients with generalized seizures, the seizure type was classified as unclassified generalized (GSE); in these patients, focal onset could not be confirmed. Complex partial SE (CPSE) included seizures where consciousness was impaired, with or without motor signs. Because of the inclusion criterion that the study patients were first treated in the ED, there were no patients with postresuscitation myoclonic jerks.

### Etiology of SE

The etiology of the SE was classified as cerebrovascular disease, brain tumor, traumatic brain injury (TBI), CNS infection, alcohol, structural lesion, other, or unknown. The majority of the patients with epilepsy used newer AEDs that do not require monitoring of blood drug levels. Furthermore, there was no definitive evidence that low drug levels were the cause of SE in any of the study patients. For these reasons, we could not consider low blood concentrations of an AED to be the main underlying cause of SE in any of the study patients. We categorized the underlying causes of SE as acute symptomatic, remote symptomatic, probably symptomatic, or idiopathic depending on the patient history and the results of the neuroimaging and lumbar puncture, when performed.

### Imaging studies and electroencephalograph (EEG)

We analyzed the results of acute neuroimaging [brain computer tomography scanning (CT) or magnetic resonance imaging (MRI)] and the findings of the 21-channel emergency EEG, when performed.

### Medication

Medications taken by the patients in the study included levetiracetam (13 patients), valproic acid (14 patients), carbamazepine (7 patients), oxcarbazepine (7 patients), topiramate (6 patients), lamotrigine (5 patients), pregabalin (4 patients), phenytoin (3 patients), gabapentin (1 patient), and lacosamide (1 patient). Additionally, the use of benzodiazepines, antipsychotics, antidepressants, or other medications was evaluated.

The treatment protocol for SE consisted of first- (diazepam or lorazepam), second- (phosphenytoin, valproic acid, or levetiracetam), third- (propofol, midazolam or thiopenthal), and fourth-line (topiramate) AEDs. The AED treatment was recorded, including the time from the beginning of the seizure to the initiation of the drug intervention, the treatment response to each drug, and the time from the initiation of the treatment to the end of the seizure.

### Outcome

The immediate outcome was estimated by classifying the neurological condition into one of the following groups using a slightly modified Rankin Scale, a scale used to assess level of function in neurological disorder: good recovery (no symptoms or no significant disability; able to carry out usual activities, despite some symptoms), slight disability (able to look after own affairs without assistance, but unable to carry out all previous activities), moderate disability (requires some help, but able to walk unassisted), severe disability (unable to attend to one’s own bodily needs without assistance, and unable to walk unassisted or requires constant nursing care and attention, bedridden, incontinent), death, or unknown [8,9]. Any relapses of seizures in the hospital, as well as later relapses during the study period, were recorded.

### Data analysis

Statistical differences between categorical variables were tested using Pearson’s chi-squared test. A binomial logistic regression model was used to evaluate the prognostic value (OR) of the categorical factors to the outcome and later seizure recurrence. The treatment delays were analyzed as non-parametric variables and were presented as median values. SPSS 17.0 was used for statistical analysis.

## Results and discussion

### Results

The clinical characteristics of the study patients are presented in Table 1. Females comprised 45% of the patients; the mean age was 62.6 years (range 17–89 years). In the patient population, 43% had epilepsy, and 32% has previous episodes of SE. In addition, 32% had cerebrovascular disease, 4.5% had dementia, 3.7% had a brain tumor, and 1.8% had a CNS infection.

**Table 1:** Clinical characteristics of the study cases

	**Total**	**Percent**
Brain trauma		
New	6	5.5
Old	10	9.2
Seizure etiology		
Acute symptomatic	39	35.8
Remote symptomatic	54	49.5
Probably symptomatic or idiopathic	16	14.7
Type of seizure		
Unclassified GSE	19	17.4
SGSE	54	49.5
SPSE	2	1.8
CPSE	31	28.4
MSE	1	0.9
Not known	2	1.8
The beginning of the SE		
Observed	23	21.1
Found	38	34.9
Unknown	48	44.0
EEG		
Generalized epileptiform activity	11	10.0
Focal epileptiform activity	22	20.2
General slowing	18	16.5
Focal slowing	14	12.8
Normal	4	3.7
Not performed	40	36.7
Discharge condition		
Normal	21	19.2
Minor disability	29	26.6
Moderate disability	33	30.3
Severe disability	18	16.5
Deceased	6	5.5
Unknown	2	1.8
Total	109	100
Time in hospital (days, mean/range)	8.06/range 2–31	
Intubation	23	21
Late recurrence		
Same period	1	0.9
Later	31	28

GSE, generalized status epilepticus; SGSE, secondarily generalized status epilepticus; SPSE, simple partial status epilepticus; CPSE, complex partial status epilepticus; MSE, myoclonic status epilepticus; EEG, electroencephalograph. Acute symptomatic etiology of seizure means condition with acute cerebral insult with seizures.

### Treatment delays and outcome

**Figure 1:**
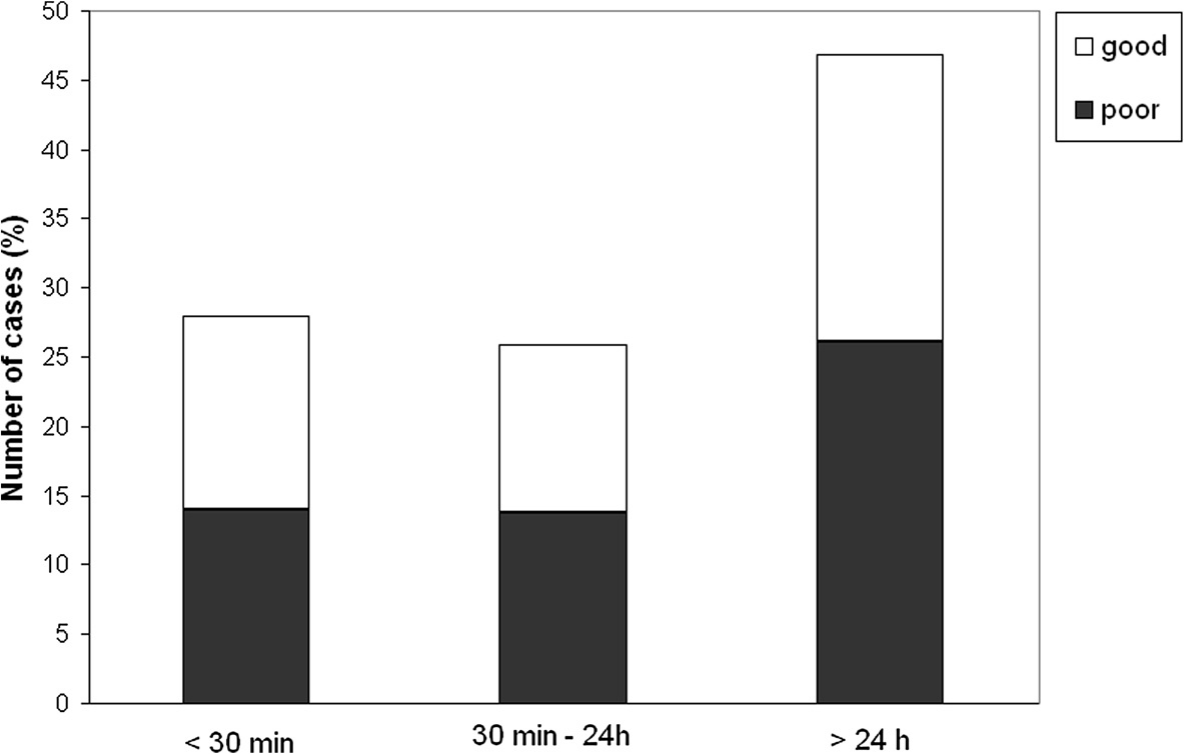
**The delay of paramedic arrival and patient outcome. **Outcome was classified as good (normal or minor disability) or poor (moderate or severe disability or death).

**Figure 2:**
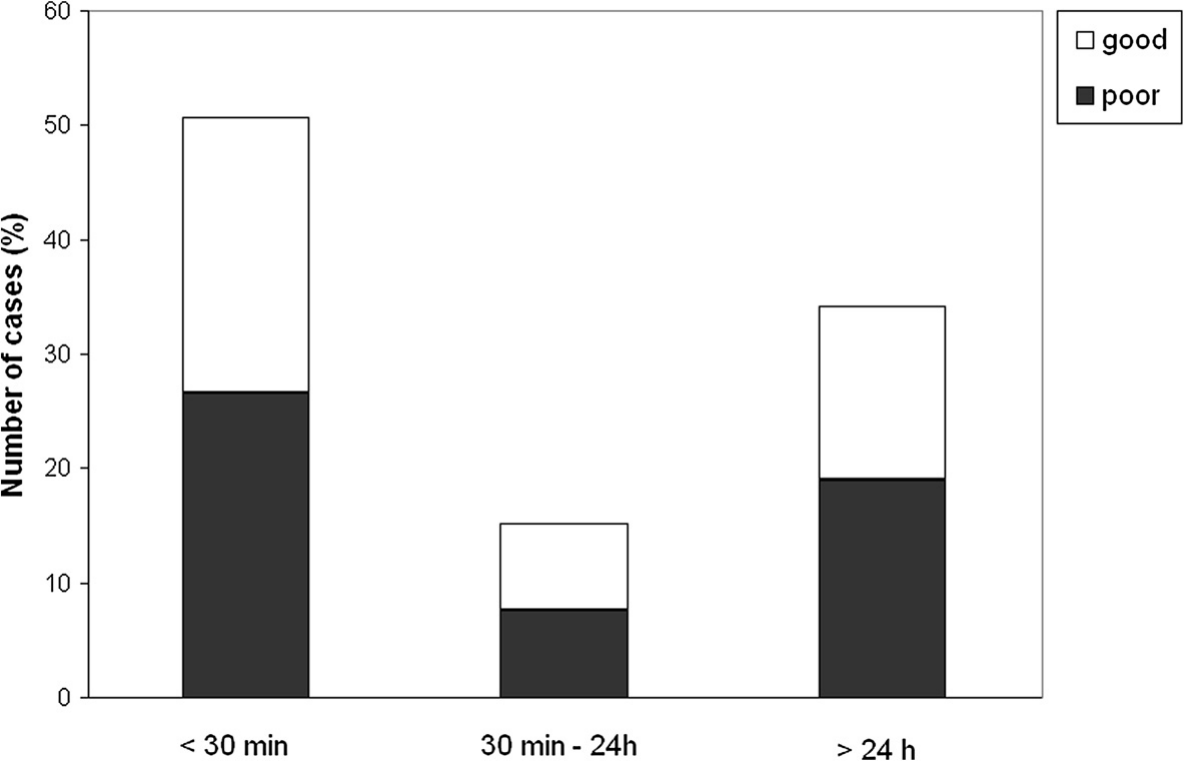
**The delay in out-of-hospital AED initiation and patient outcome. **Outcome was classified as good (normal or minor disability) or poor (moderate or severe disability or death).

**Figure 3:**
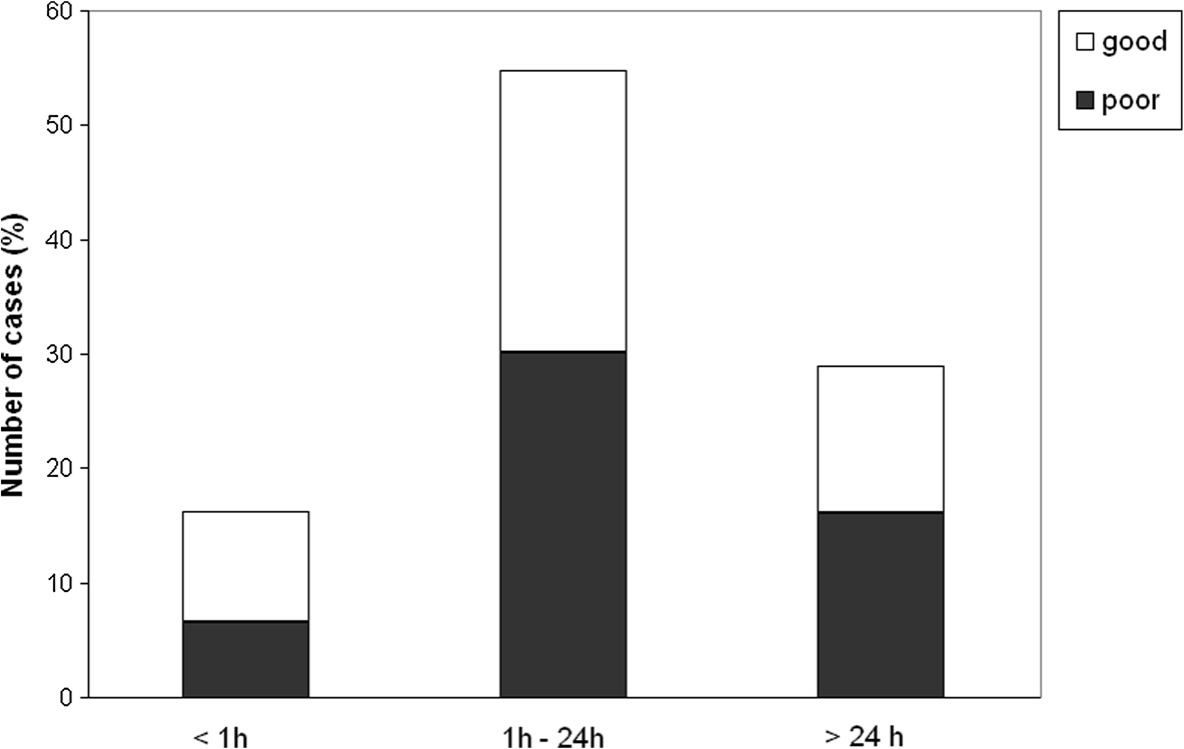
**The delay in arrival at the ED and patient outcome. **Outcome was classified as good (normal or minor disability) or poor (moderate or severe disability or death).

**Figure 4:**
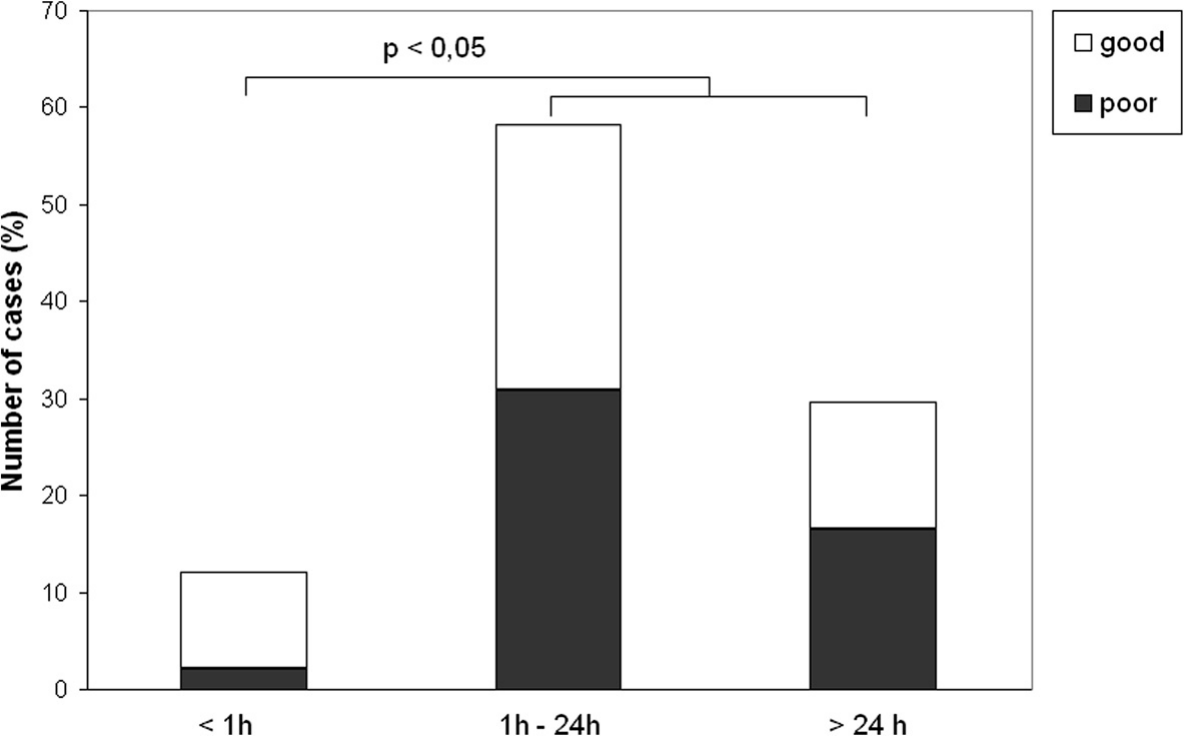
**The delay in AED initiation and patient outcome. **Outcome was classified as good (normal or minor disability) or poor (moderate or severe disability or death).

The median delay for paramedic arrival was 30 min, initiation of out-of-hospital AEDs 1 h 10 min, arrival at the ED 1 h 45 min, and initiation of AED in the ED 2 h 11 min. To evaluate the effect of treatment delays on outcome, patients were classified according to delays as follows: arrival of paramedics and initiation of out-of-hospital AEDs in (1) less than 30 min, (2) 30 min to 1 h, or (3) more than 1 h (up to 96 hours). The delay between arrival at the ED and initiation of AEDs in the hospital was classified as follows: (1) less than 1 h, (2) greater than 1 h but less than 24 h, or (3) 24 h or more (up to 96 h). The outcome was classified as follows: good (normal or minor disability) or poor (moderate or severe disability or death). The distribution of patients in these groups according to outcome is presented in Figures 1, 2, 3, and 4.

We found no significant differences in the patient outcome with respect to treatment delays (Figure 1), except that patients for whom emergency AED treatment was initiated in the ED within 1 h of seizure onset showed good recovery in 82% (9 out of 11) of cases, whereas only 46% of patients for whom AED treatment was initiated more than 1 h after the seizure onset had a good outcome (*p* < 0.05, Pearson’s chi-square test).

### Categorical factors predicting outcome

The etiology of the seizure was cerebrovascular disease in 41.2%, alcohol misuse in 18.3%, trauma in 5.5%, infection in 5.5%, tumor in 4.6%, structural anomaly in 1.8%, other in 16.5%, and unknown in 6.4% of the cases. We investigated the effect of clinical variables on the outcome by categorizing the outcome condition into two classes: good (normal or minor disability) or poor (moderate or severe disability or death). The presence of cardiovascular disease was associated with a poor outcome (OR 2.28, 95% CI: 1.04–4.96; *p* = 0.039); 36% of the patients with cardiovascular disease had a good outcome, and 64% had a poor outcome (compared to 56% and 44% of patients without cardiovascular disease, respectively). Epileptiform activity in an EEG examination was associated with a poor outcome (OR 3.00, 95% CI: 1.11–8.1; *p* = 0.028). For further analysis, we grouped the etiology of SE into three categories: cerebrovascular disease, alcohol misuse, and other. No statistical differences in outcome were found between the groups. Furthermore, there were no differences between generalized seizure and other seizure types with respect to the outcome. Interestingly, whether the beginning of the seizure was observed was not associated with disability.

### Categorical factors predicting seizure recurrence

A history of epilepsy was associated with the rate of seizure recurrence (OR 3.37, 95% CI: 1.41–8.05; *p* = 0.006). Additionally, a history of SE was associated with the risk of seizure recurrence (OR 6.04, 95% CI: 2.43–14.97; *p* < 0.001). Alcohol abuse seemed to increase the risk of recurrence (OR 3.765, 95% CI: 1.55–9.14; *p* = 0.003), but it did not have an effect on disability; the outcome was good in 35% and poor in 65% of the cases (*n* = 20). The presence of diabetes or CNS disease did not have an effect on disability.

### Medication

Benzodiazepine given out of hospital did not have an effect on the outcome or rate of seizure recurrence. Epileptiform activity in the EEG was more common among those who did not get benzodiazepines out of hospital; there were discharges in the EEG of 41% of patients in the benzodiazepine group and 56% of patients in the non-benzodiazepine group, but the difference was not statistically significant. The type of AED given first (lorazepam, diazepam, or phosphenytoin) in the ED did not seem to associate with findings in the EEG, the outcome, or seizure recurrence rate. The proportion of AEDs given in the ED was as follows: lorazepam, 59 patients; diazepam, 26; phosphenytoin, 72; levetirasetam, 6; valproic acid, 3; oxcarbazepine, 1; and propofol, 11.

Findings in brain CT examinations, the type of seizure, the etiology of SE, and the general condition of the patient in the out-of-hospital period were not associated with the outcome. The GCS before the patient was brought into the ED did not have an effect on the disability or later relapses. Six patients died in the hospital during the SE period. Three of those patients had an acute stroke, one had Down syndrome and epilepsy, one had misused alcohol, and one had an acute myocardial infarction.

### Discussion

Current guidelines for treating SE emphasize immediate action to interrupt the seizure. However, in real life, several variables interfere with optimal treatment. The treatment delay in this study was surprisingly high in both patients with known, but specifically with unknown, duration of the seizure. Regardless, the delays were not associated with the disability of the patients. This surprising finding is partly explained by the fact that all of the patients had a severe condition. It is worth noting that a remarkable proportion of the patients (56 cases) did not get any out-of-hospital drug treatment. These patients either did not have visible convulsions or the convulsions were intermittent. When evaluating the group with a known duration of the seizure, the treatment delays were shorter but still quite long. The delay is at least partly explained by the long distances between hospitals and the populations that they serve in Finland. Due to several factors, it was impossible to quantify the exact duration of SE. The retrospective nature of this study made it difficult to determine the duration of the SE based on medical records. Even in the clinical setting, determination of the exact time to recovery and the treatment response is challenging, especially in patients with NCSE.

We analyzed several clinical characteristics that are important to accurately evaluate the acute outcome of patients with SE. From a clinical point of view, the common opinion that a primary or secondary generalized seizure has a more severe influence on recovery was not confirmed in the present study. Manifestations of SE are highly diverse, and outcomes are influenced by different variables. Epileptiform activity in the EEG was associated with a poor outcome, which supports the importance of an EEG examination in the ED. With this in mind, an EEG could be regarded as the most important prognostic biomarker when evaluating the prognosis of a patient with SE. This finding supports the need for an immediate EEG evaluation in all patients with SE [10].

The etiology of SE did not have an effect on the outcome. The two major etiologies in this study were cerebrovascular disease and alcohol misuse. Both acute symptomatic and remote symptomatic seizures caused by stroke are known to be associated with an increased risk of long-term mortality [11]. The immediate prognosis of SE, and especially GCSE, seems to be poor in post-stroke epilepsy [12,13]. The misuse of alcohol, especially during withdrawal from drinking, is a common problem in the ED. The prognosis of these patients depends on other risk factors, such as TBI and metabolic problems. External toxicological agents, the elimination of which may improve prognosis, primarily cause pure withdrawal seizures. In a study of 249 patients with GCSE, alcohol was the major cause of SE in 10.8% of the patients [14]. The overall prognosis was favorable, although there were patients with prolonged postictal state. The sample size was not sufficient to answer the question about the variables affecting the outcome of different disease groups. However, it is possible that those patients with alcohol-related seizures had underlying although unknown precipitating factors worsening the prognosis. In addition, we did not have a possibility to evaluate the long-term prognosis of these patients.

The small proportion of patients with TBI in the present study is explained by the fact that, in TBI patients, the SE is usually a secondary complication of the primary disorder. We did not find patients with low AED blood concentrations in this study. This problem as an underlying cause of SE is difficult to prove because the majority of epilepsy patients use new AEDs, which do not require monitoring of the blood concentrations. However, the measurement of serum drug levels in the treatment of SE would be important in evaluating the possibility of non-compliance and other causes of inadequate AED dose. In a previous study, the long-term mortality rate was higher in patients with acute symptomatic SE, but that study differed from ours because it included patients with myoclonic SE after anoxic encephalopathy [15]. In 15% of the patients, the underlying etiology of SE was regarded as probably symptomatic or idiopathic, which matches the findings in the Minnesota study (17.5%) [16]. We included only patients with a diagnosis of SE upon admission to the hospital but not patients with anoxia. The existence of cardiovascular disease was associated with a worse outcome: two-thirds of the patients with cardiovascular disease had poor outcome. This finding supports the use of a holistic perspective in evaluating patients in the acute setting.

The impact of the first-line treatment in the out-of-hospital stage was surprisingly small. There were no differences in disability or later relapses between the patients with or without treatment at the out-of-hospital stage. However, in all of the study patients, SE was treated very aggressively immediately upon arrival at the ED. We did not find any differences between the various first-line AEDs administered in the ED. In the present study, there were no patients receiving intramuscular midazolam, which has proved effective compared with intravenous administration of lorazepam [17].

There are some weaknesses of this study. The small proportion of epilepsy patients with prolonged seizures is diagnosed only with their chronic epilepsy diagnosis number. Missing data on delays weaken the applicability of the results; however, very long delays in a proportion of patients warrants the need for development of the emergency medicine system. We included only patients with SE at admission to the ED, not those patients with SE as a complication of their primary disease leading to the treatment in the hospital. As a result, this study concentrates on the treatment of the SE in the ED.

## Conclusions

To conclude, this study demonstrates which clinical factors are important in evaluating patients with SE in the ED. Treatment delays could be shortened by increasing paramedics’ understanding and knowledge of atypical SE and by improving the quality of emergency medicine services.

## Competing interests

Suvi Liimatainen has served as a paid consultant for UCB Pharma and has received support from GlaxoSmithKline and UCB Pharma. Jukka Peltola has served as a paid consultant for Eisai, Jazz Pharma, and UCB Pharma and has received support from Cyberonics, Eisai, Medtronics, Orion Pharma, and UCB Pharma. For the remaining authors, no conflicts were declared.

## Authors’ contributions

JH participated in the design of the study, collected the data, and wrote the manuscript. KL performed the statistical analysis and wrote the manuscript. JP participated in the design of the study and helped to draft the manuscript. SL participated in the design of the study, coordinated the it, and wrote the manuscript. All authors read and approved the final manuscript.
